# Distribution and morphology of sensory and autonomic fibres in the subendocardial plexus of the rat heart

**DOI:** 10.1111/joa.13284

**Published:** 2020-08-11

**Authors:** Fiona C. Shenton, Thomas Campbell, James F. X. Jones, Susan Pyner

**Affiliations:** ^1^ Department of Biosciences Durham University Durham UK; ^2^ Discipline of Anatomy School of Medicine University College Dublin Dublin 4 Ireland

**Keywords:** atrial mechanoreceptors, atrial volume receptors, calcitonin gene‐related peptide, choline acetyltransferase, complex unencapsulated endings, end‐net, flower‐spray endings, mechanotransduction, methylene blue, neurofilament, plasma volume regulation, synaptic vesicle protein 2, synaptophysin, tyrosine hydroxylase

## Abstract

Cardiac reflexes originating from sensory receptors in the heart ensure blood supply to vital tissues and organs in the face of constantly changing demands. Atrial volume receptors are mechanically sensitive vagal afferents which relay to the medulla and hypothalamus, affecting vasopressin release and renal sympathetic activity. To date, two anatomically distinct sensory endings have been identified which may subserve cardiac mechanosensation: end‐nets and flower‐spray endings. To map the distribution of atrial receptors in the subendocardial space, we have double‐labelled rat right atrial whole mounts for neurofilament heavy chain (NFH) and synaptic vesicle protein 2 (SV2) and generated high‐resolution maps of the rat subendocardial neural plexus at the cavo‐atrial region. In order to elucidate the nature of these fibres, double labelling with synaptophysin (SYN) and either NFH, calcitonin gene‐related peptide (CGRP), choline acetyltransferase (ChAT) or tyrosine hydroxylase (TH) was performed. The findings show that subendocardial nerve nets are denser at the superior cavo‐atrial junction than the mid‐atrial region. Adluminal plexuses had the finest diameters and stained positively for synaptic vesicles (SV2 and SYN), CGRP and TH. These plexuses may represent sympathetic post‐ganglionic fibres and/or sensory afferents. The latter are candidate substrates for type B volume receptors which are excited by stretch during atrial filling. Deeper nerve fibres appeared coarser and may be cholinergic (positive staining for ChAT). Flower‐spray endings were never observed using immunohistochemistry but were delineated clearly with the intravital stain methylene blue. We suggest that differing nerve fibre structures form the basis by which atrial deformation and hence atrial filling is reflected to the brain.

## Graphical abstract

In search of atrial volume receptors, we performed immunohistochemical characterisation of the subendocardial nerve plexus of the rat right atrium. Rats possess a bicuspid Eustachian valve which is richly innervated. Fine adluminal end‐nets expressed vesicular markers, CGRP and tyrosine hydroxylase. Immunohistochemistry did not label flower‐spray endings but these were identified with methylene blue. The observed plexuses could represent sympathetic post‐ganglionic and/or sensory afferents.

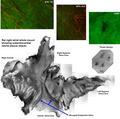

## 1 INTRODUCTION

Important cardiac reflexes help to ensure that the blood supply to all tissues and organs is maintained in the face of constantly changing demands. Venous return to the right atrium influences the measured variable of the atrial volume reflex. Returning blood stimulates cardiac mechanoreceptors located at the cavo‐atrial junction which in turn signal to cardiac control centres in the brain and moderate sympathetic drive to the heart and kidney. Atrial filling and contraction produce structural deformation in three dimensions, and therefore, cardiac mechanoreceptors must accurately reflect the deformation. Atrial receptors form a mechanically sensitive family of afferent parasympathetic neurons whose sensory endings are primarily concentrated within the endocardium at the cavo‐atrial junctions of the heart (Woollard, 1926; Coleridge *et al.*, 1957; Holmes, 1957a; Tranum‐Jensen, 1975). However, the morphological nature of the mechanoreceptor/sensory nerve ending type underpinning this important function has not been unequivocally determined.

Despite detailed histological studies of sensory nerve endings in the mammalian heart, there still remains some disagreement concerning the types and distribution of these structures. Silver impregnation (Nonidez, 1937) and methylene blue staining (Holmes, 1957a; 1957b; Miller and Kasahara, 1964) indicate that the atrial endocardium contains cardiac mechanoreceptors (see Linden and Kappagoda, 1982 for review). Two anatomically distinct nerve endings have been described as candidate sensory specialisations for atrial receptors: end‐nets and complex unencapsulated endings (diffuse and compact) or flower‐sprays. End‐nets are described as large arrays of anastomosed dendrites and flower‐sprays as arborised complex unencapsulated endings. The morphology of end‐nets and flower‐sprays has been well described with methylene blue labelling in dogs, cats, monkeys, rabbits and guinea pigs (Coleridge *et al.*, 1957; Miller and Kasahara, 1964; Linden and Kappagoda, 1982). A third type, complex encapsulated ending has also been described but is thought to be rare in mammals (see Hainsworth, 1979 for review). Cheng and colleagues, in the rat, labelled vagal afferents with DiI (1,1′‐dioleyl‐3,3,3′3′‐tetramethylindocarbocyanine methanesulfonate) and identified “flower‐spray” or complex unencapsulated endings and “end‐net” terminals (Cheng *et al.*, 1997). It has been suggested that the morphological differences that appear to exist between unencapsulated endings and end‐nets are quantitative rather than qualitative and that end‐nets should in fact be considered as extensive unencapsulated endings (Hainsworth *et al.*, 1979).

For complex unencapsulated endings, it is reported the atria contain more of these than the endocardium of any other part of the heart and only the atria contain these endings and end‐nets. The end‐net structure has been described as distinct from that of the complex unencapsulated end organs (Coleridge *et al.*, 1957; Holmes, 1957a; 1957b; 1958) and suggests this structure is present in both atrial and ventricular endocardium but more abundant in the atria (Miller and Kasahara, 1964). Notwithstanding the discrepancies about receptor identity, it is apparent that end‐nets and complex unencapsulated end‐organs form challenging three‐dimensional structures.

The observation that end‐net distribution is more extensive throughout the atrial endocardium than the complex unencapsulated nerve endings (Coleridge *et al.*, 1957; Holmes, 1957b) may be important in terms of signalling atrial function to the brain. One suggestion is that receptor discharge is related to the force exerted on the atrial wall due to ventricular contraction (Langrehr, 1960). Another theory argues that the activation of the receptors is a function of atrial wall tension itself and is best described by the Law of Laplace (Kidd *et al.* 1966, 1978). Nevertheless, it is apparent that during atrial contraction parts of the atria undergo extensive mechanical distortion. The question arises: what is the optimal type of network to convey this information faithfully to the brain?

Therefore, to understand the anatomical basis of receptor morphology and density we mapped the distribution of atrial receptors in the subendocardial space of the rat heart. Immunohistochemistry was carried out on right atrial whole mounts for neurotransmitters/proteins/markers to enable discrimination between sensory and motor (autonomic) axons to reveal putative cardiac receptor morphology.

Early findings relating to NFH/SV2 double labelling have been presented in preliminary form at the Physiological Society annual conference – Proc Physiol Soc 43 (2019) C102.

## 2 MATERIALS AND METHODS

### 2.1 Ethical approval

All experiments were approved by the Local Ethics Committee of Durham University and University College Dublin (AREC‐15‐36‐Jones) and performed in accordance with United Kingdom (UK) Animals (Scientific Procedures) Act, 1986 and the European Commission Directive 2010/63/EU (on the protection of animals used for scientific purposes).

### 2.2 Atrial tissue preparation

Atrial tissue was prepared using different methodologies at two institutions (Dublin and Durham). For method one (Dublin), Wistar rats (*n* = 15, 140–400 g) were euthanised by cervical dislocation whilst under inhalational anaesthesia (5% isoflurane). Thereafter, a median sternotomy was performed, heparinised saline was injected into the cavity of the right ventricle, and exposed mediastinal tissue was briefly perfused with oxygenated Tyrode’s solution. The heart was removed and placed in oxygenated Tyrode’s solution (20°C). Coronary circulation flushing with Tyrode’s solution through the ascending aorta removed blood. The right atrium and great veins were isolated, and the right atrium was oriented such that the lumen of the cavity was exposed. An incision was made along the long axis of the inferior and right superior vena cava. The tissue was pinned flat to expose the endocardial surface. The whole mount was then fixed and permeabilised in precooled methanol (Sigma‐Aldrich, 34860) at −20°C for 30 min. Squashed and teased fibres of hind feet fourth lumbrical muscles were similarly prepared and utilised as positive control tissues for NFH and SV2 investigations. For method two (Durham), Wistar rats (*n* = 32, 150–200 g) were terminally anaesthetised with an overdose of sodium pentobarbital (60 mg/kg) and perfused with heparinised saline followed by 4% (w/v) paraformaldehyde in 0.1 M phosphate buffer (PB; pH 7.4). Following a median sternotomy, the heart was removed and post‐fixed overnight at 4°C. The following day the hearts were removed from fixative and rinsed in 0.1 M PB. The right atrium and the entrances of the three caval vessels: left and right superior vena cavae (L‐ and R‐SVC) and inferior vena cava (IVC), were isolated and opened out to expose the lumenal surface, to give a whole mount preparation.

### 2.3 Immunohistochemistry

Whole mounts obtained by method one were blocked with 1% (w/v) bovine serum albumin (BSA; Sigma‐Aldrich, A2153), 5% (v/v) normal goat serum (Sigma‐Aldrich, G9023) and 1X phosphate‐buffered saline (PBS, Gibco). Primary antibody diluent was prepared in blocking solution, and subsequently, incubation was performed overnight in a humidity chamber at 4°C. The primary antibodies were anti‐SV2 and anti‐NFH (see Table 1). Following primary incubation, samples were triple rinsed with 1% BSA (w/v) in PBS (1×) with each rinse having a duration of 10 min. Tissue was subsequently incubated overnight in a light‐protected humidity chamber at 4°C with secondary antibody diluted in 1% (w/v) BSA and PBS (1×). Prior to imaging, all samples were triple rinsed in PBS (3 × 10 min duration), mounted in OCT on polysine slides and cover slipped. Whole mounts obtained by method two were washed briefly in 0.1M PB. Non‐specific binding sites were blocked with 10% (v/v) normal donkey serum (NDS, Abcam, Ab7475). Subsequently, whole mounts were permeabilised in Triton X‐100 (0.1% v/v in PB) for 45 min, rinsed in PB (1 × 10 min) and then incubated in primary antibody for 48 hr at 4°C. The primary antibodies were a combination of one of the four test antibodies (anti‐CGRP, anti‐NFH, anti‐ChAT or anti‐TH) together with the marker antibody, either mouse or rabbit anti‐SYN as appropriate. The antibody diluent for both the primary and secondary antibodies was PB containing 1% NDS. At least six animals were used for each combination. For details of all primary and secondary antibodies, see Table 1. After washing (×3 in PB), secondary antibodies were applied for two hours at room temperature in the dark. Finally, whole mounts were washed as before and spread onto charged slides (Superfrost Plus; Fisher, 10149870). After air drying overnight, whole mounts were dehydrated through a series of alcohols (2 min in 50%, 70%, 95% and 100%), cleared in xylene (4 min), mounted in DPX and cover slipped. All of the described secondary antibodies were fluorescent conjugates. These protocols are presented as flow diagrams in the Appendix S1 section. Different vesicular markers (SV2 or SYN) were used at each institution because each research team had prior experience with vesicle labelling (SV2 at UCD and SYN at Durham University), and both markers were expected to produce similar labelling.

**Table 1:** Table of antibodies

Antibody	Immunogen	Source	Dilution
Mouse ⍺ SYN	Presynaptic vesicles	Abcam Cat# ab8049, Mouse monoclonal [SY38] to synaptophysin RRID:AB_2198854	1:200
Rabbit ⍺ SYN	Synthetic peptide corresponding to human synaptophysin aa 41–62, Sequence: FATCGSYSGELQLSVDCANKTE	Abcam Cat# ab14692, Rabbit polyclonal to synaptophysin RRID:AB_301417	1:200
Mouse ⍺ NFH	Recombinant full‐length protein corresponding to human neurofilament heavy polypeptide aa 1–1026	Abcam Cat# ab187374, Mouse monoclonal [NF421] to neurofilament heavy polypeptide (RRID Unavailable)	1:200
Mouse ⍺ CGRP	Rat alpha‐CGRP	Abcam Cat# ab81887, Mouse monoclonal [4901] to CGRP RRID:AB_1658411	1:80
Goat ⍺ CGRP	Synthetic peptide corresponding to Rat CGRP (C terminal), Sequence: VKDNFVPTNVGSEAF	Abcam Cat# ab36001, Goat polyclonal to CGRP RRID:AB_725807	1:80
Rabbit ⍺ TH	Full‐length SDS denatured protein (purified from pheochromocytoma) (Rat)	Abcam Cat# ab112, Rabbit polyclonal to tyrosine hydroxylase RRID:AB_297840	1:1,000
Goat ⍺ ChAT	Human placental enzyme	Millipore Cat# AB144P, Goat polyclonal to choline acetyltransferase RRID:AB_2079751	1:80
Chicken ⍺ NFH	Full‐length protein corresponding to cow neurofilament heavy polypeptide	Abcam Cat# ab72996, RRID:AB_2149618	1:500
Mouse ⍺ SV2	Purified synaptic vesicles	Developmental Studies Hybridoma Bank (DSHB) Cat# SV2 RRID:AB_2315387	1:25
Donkey anti‐mouse IgG Alexa Fluor 488		Thermo Fisher Cat# A−21202, RRID:AB_141607	1:200
Donkey anti‐rabbit IgG Alexa Fluor 488		Thermo Fisher Cat# A−21206, RRID:AB_2535792	1:200
Donkey anti‐goat IgG Alexa Fluor 488		Thermo Fisher Cat# A−11055, RRID:AB_2534102	1:200
Donkey anti‐mouse IgG Alexa Fluor 594		Thermo Fisher Cat# A−21203, RRID:AB_2535789	1:200
Donkey anti‐rabbit IgG Alexa Fluor 594		Thermo Fisher Cat# A−21207, RRID:AB_141637	1:200
Goat anti‐chicken IgY Alexa Fluor 568		Abcam Cat# ab175477, (RRID unavailable)	1:250
Goat anti‐mouse IgG‐FITC		Sigma‐Aldrich Cat# F8771, RRID:AB_259778	1:100

### 2.4 Image acquisition and analysis

Immunohistochemical samples labelled with anti‐NFH and/or anti‐SV2 were examined under epifluorescence using an Olympus BX51 microscope fitted with Texas Red and FITC filters. Images were captured using an Olympus DP71 camera. Olympus cell Sens imaging software (Standard 1.15, Build 14760) was used to display and save images. For NFH whole mount mapping, the entire endocardial surface was imaged manually with a ×4 objective, allowing approximately 15% overlap between adjacent images to minimise difficulty during image stitching. Whole mount montages were created using the pairwise stitching plugin in Fiji (ImageJ 1.52i) (Preibisch *et al.*, 2009). Stitched montages were converted to 8‐bit greyscale, colour‐inverted and contrast enhanced within Fiji (ImageJ v1.52i). The tissue labelled with anti‐SYN, anti‐NFH, anti‐CGRP, anti‐TH or anti‐ChAT was examined using a Zeiss Axioskop 2 under epifluorescence (Texas Red and FITC filters). Digital images were captured with a Hamamatsu Orca 285 CCD camera controlled by Improvision Volocity (Acquisition, Restoration and Visualisation) software (v. 6.2.1). Images were captured for analysis at superficial/deep endocardial and subendocardial‐myocardial planes. Final images were imported into Adobe Photoshop Creative Cloud (v20.0.4) to create annotated figures.

### 2.5 Stereological analysis of subendocardial neural plexus density

**Figure 1:**
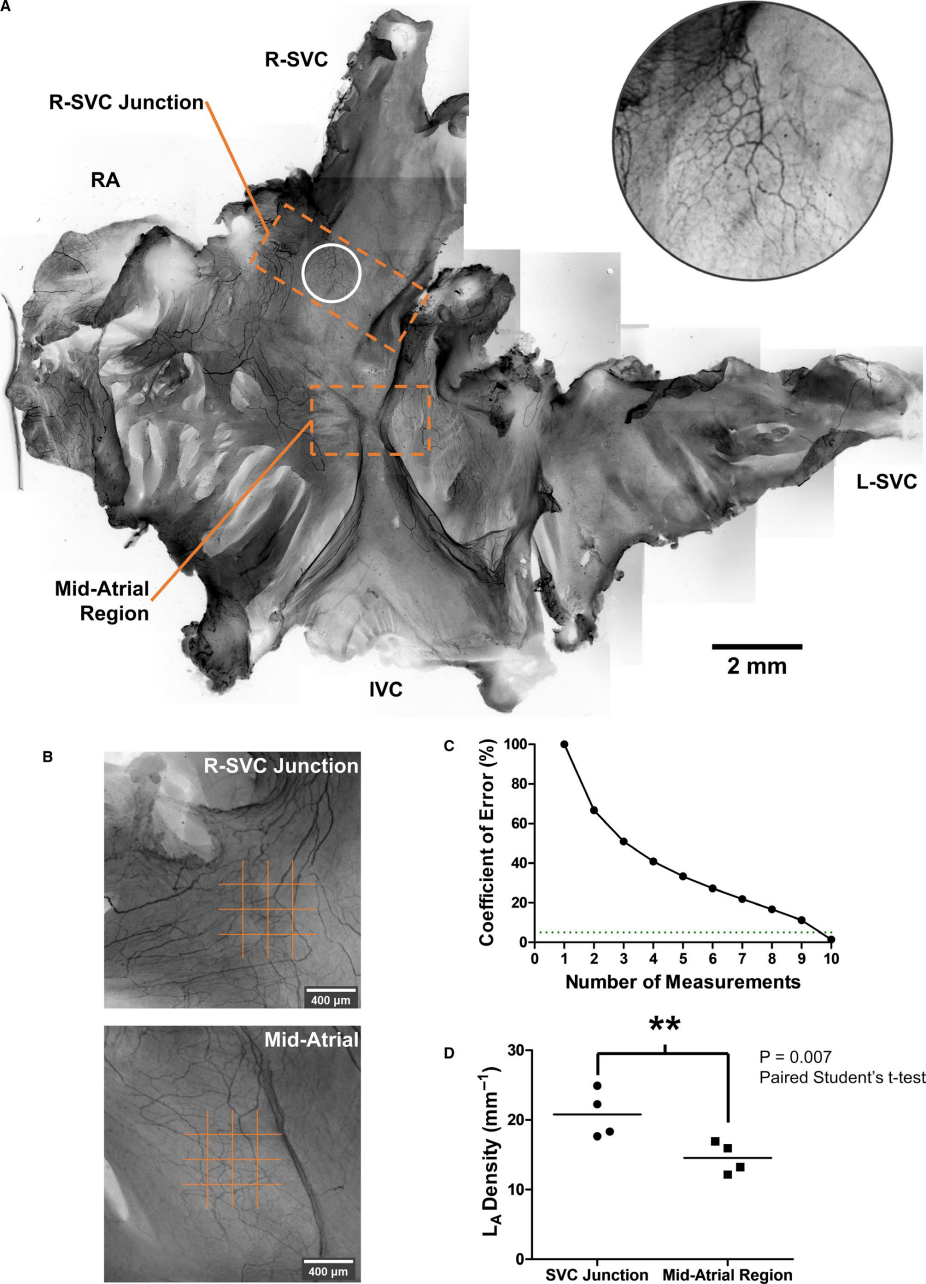
NFH labelling of the subendocardial neural plexus in the right atrium of the rat. (a) Montage of right atrial whole mount with neurofilament labelling (black) with an inset white circle showing an end‐net arrangement of axons. (b) Stereological probe (orange) used to measure innervation density. Each limb of the probe was 800 μm in length. (c) Coefficient of error for repeat measures with stereological probe. Dotted line represents 5% coefficient of error. (d) Innervation density (*L*_A_ Density) comparison between the superior cavo‐atrial junction and the mid‐atrial region. IVC, inferior vena cava. L‐SVC, left superior vena cava; NFH, neurofilament heavy chain; RA, right auricle; R‐SVC, right superior vena cava

Subendocardial neural plexus density measurement was performed using atrial whole mounts prepared by method one. A digital stereological probe was constructed and consisted of six linear probes (3 × 3) with each linear probe having a length of 800 µm (see Figure 1b). Parallel linear probes were set 200 µm apart, and the total area covered by the stereological probe measured 800 µm × 800 µm. For efficient sampling precision, the coefficient of error for a stereological probe was calculated (see Appendix S1). The probe was placed at a specific location in an image of superior cavo‐atrial junction and underwent a randomised rotation. The total number of axon‐probe intersections was counted, and the coefficient of error was calculated. This process was repeated until the coefficient of error was below 5% which informed the number of repeat measures required for each region of the subendocardial neural plexus in our assessment of innervation density. The density of the subendocardial neural plexus was determined by measuring the length area density (*L*_A_) per unit area (Mouton, 2011). Details regarding this calculation are provided in the Appendix S1. The length of the network for a region of interest was estimated as the number of network intersections created with a linear probe of known length. Linear probes were placed as a series of parallel lines with equidistant spread. Horizontal and vertical probes were overlaid on regions of interest of the subendocardial neural plexus. A count was performed of the total number of times an axon crossed a probe, and subsequently, this number was divided by the total length of the probe. Total probe length was the sum of horizontal and vertical probes.

### 2.6 Antibody concentrations and specificity

Table 1 details primary and secondary antibody dilutions and suppliers. In terms of specificity, these are all commercial antibodies subject to routine quality assurance. Where positive results were obtained, the pattern of reactivity was characteristic of that particular antibody with distinct cell populations consistently labelled by that antibody on repeat assays. There was an absence of labelling with secondary antibodies alone.

### 2.7 Methylene blue labelling and imaging

Isolated whole mounts of Wistar rat right atrium (*n* = 11) were prepared as per method one, that is pinned flat (endocardial surface up) on silicone and perfused in oxygenated (100% O_2_) Tyrode’s solution. The tissue was incubated in 0.2% (w/v) methylene blue (Sigma‐Aldrich, M9140) in gassed Tyrode’s solution (100% O_2_) for 25 min at 20°C. The whole mounts were then fixed (1 hr) in a precooled (4°C) solution of ammonium molybdate (6% w/v, Sigma‐Aldrich, 09878, pH 5.2) followed by rinsing with PBS (10 min) and then mounted in OCT on polysine slides and cover slipped. Samples were imaged by brightfield microscopy, using either an Olympus BX51 or a standard stereoscope. Methylene blue labelled structures were most clearly visualised with transillumination. Images were converted to 8‐bit greyscale and contrast enhanced within Fiji (ImageJ v1.52i).

## 3 RESULTS

### 3.1 Neurofilament density mapping in right atrial whole mount

**Figure 2:**
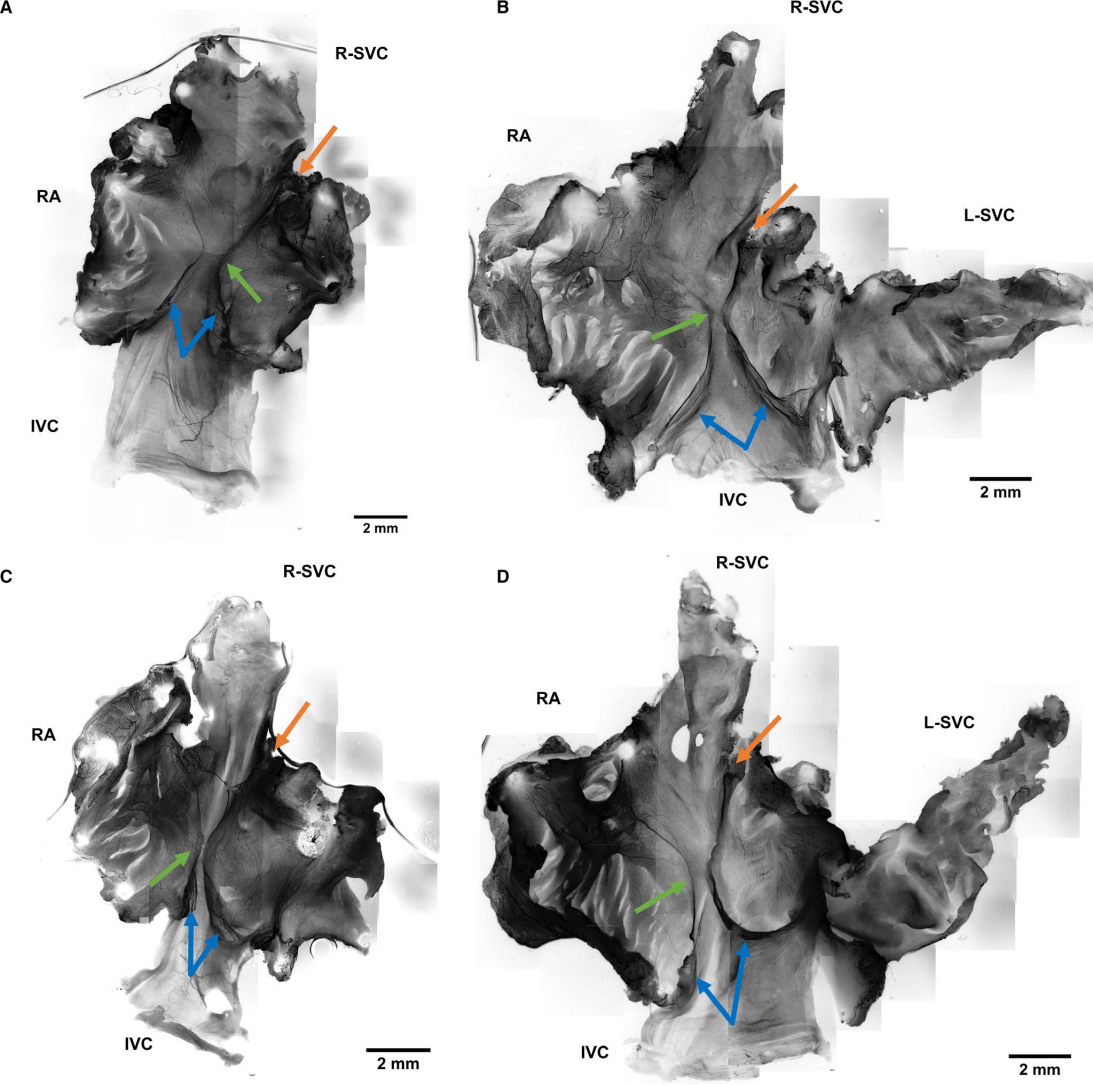
NFH labelling of right atrial whole mounts. (a–d) Neurofilament (black), labelling revealed a recurrent hourglass arrangement of axons present in the mid region of the posterior atrial wall (green arrows). The cardiac vagal branch (not shown) was noted to consistently meet the heart at the superior cavo‐atrial junction (orange arrows). The lower limbs of the hourglass correspond to the eustachian valve which is shown to be bicuspid and exquisitely innervated. (b and d) Left superior vena cava has been preserved. IVC, Inferior cava; L‐SVC, left superior vena cava; NFH, neurofilament heavy chain; RA, right auricle; R‐SVC, right superior vena cava

For the atrial whole mounts, profound axonal neurofilament labelling was noted in all samples. The right atrium was observed to be richly innervated with a neural plexus present just below the level of the endocardium. This subendocardial neural plexus was observed to be most dense at the superior cavo‐atrial junction (Figure 1). Ten measurements of *L*_A_ were required to ensure a coefficient of error <5% (CE 1.5%, *n* = 10, Figure 1c). Length area density was calculated 10 times for the superior cavo‐atrial junction and the mid‐atrial region. This was performed in four neurofilament whole mounts, totalling 80 measurements. Mean *L*_A_ for each region was calculated and compared by a paired Student's *t*‐test. Mean *L*_A_ was significantly greater at the superior cavo‐atrial region than the mid‐atrial region (*L*_A_ 20.8 ± 1.7 vs. 14.6 ± 0.5 mm^−1^, *p* = 0.007, Figure 1d). The coefficient of error for the superior cavo‐atrial region was 8.2% and 3.6% for mid‐atrial region. Thus, greater biological variation was observed at the superior junction than the mid‐atrial region (6.7% vs. 2.1%). A distinct hourglass arrangement of axons was present consistently in the posterior atrial wall (Figure 2). The limbs of this hourglass travelled to the superior and inferior cavo‐atrial junctions where they were flanked by an abundance of end‐nets. Rich innervation was also observed in the septal and auricular areas. Some innervation was observed at the junction of the left superior vena cava though it was not flanked by end‐nets. The rest of the left superior vena cava was sparsely innervated. In each montage (Figure 2), the left limb of the hourglass appeared to coincide with the crista terminalis of the right atrium. The upper right limb appeared to track towards the root of the cardiac vagal branch (Figure 2, orange arrows). Each of the lower limbs of the hourglass was suspended in a soft membranous fold (Figure 2, blue arrows) which were identified as cusps of an Eustachian valve of the IVC. Across all samples, no flower‐spray endings or varicosities resembling motor fibre terminals were observed.

### 3.2 Neurofilament and synaptic vesicle protein 2 double labelling

**Figure 3:**
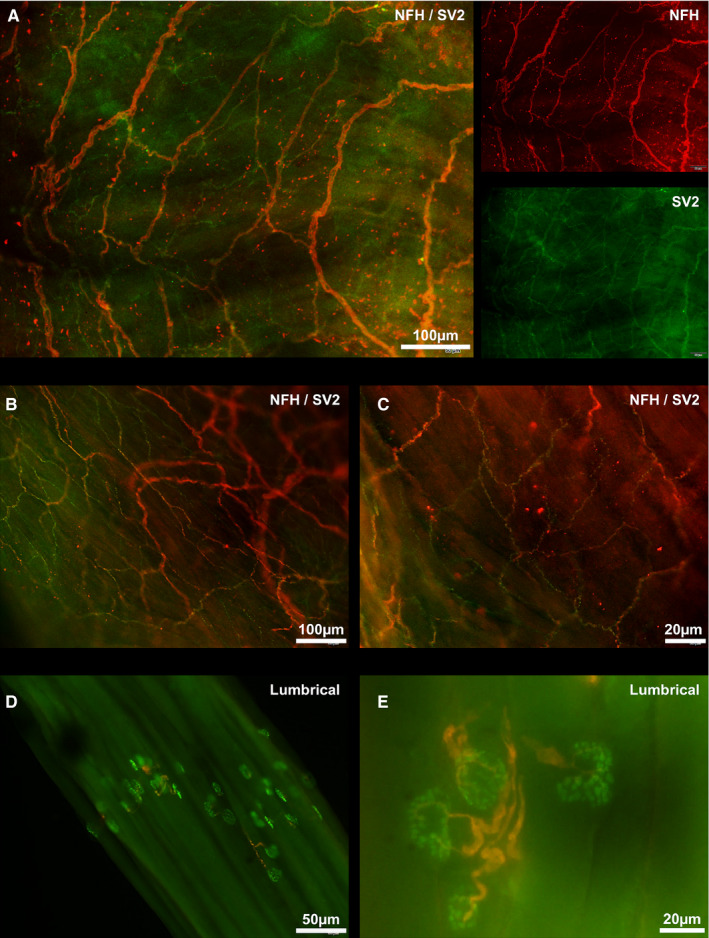
NFH and SV2 double labelling of right atrial whole mounts and lumbrical muscles. For all panels, neurofilament heavy chain (NFH) is shown in red and synaptic vesicle protein 2 (SV2) is shown in green. (a) Inferior cavo‐atrial junction end‐net with a smaller net of SV2 labelled fibres. (b) Mid‐atrial region (c) Right superior cavo‐atrial junction (d and e) NFH and SV2 labelling of teased lumbricals. Motor end plates are clearly evident. NFH labelled axons lead to axon terminals containing SV2 labelled vesicles. NFH, neurofilament heavy chain; SV2, synaptic vesicle protein 2

In atrial whole mounts, it was observed that NFH antibodies labelled large fibres whilst SV2 antibodies labelled both large and fine axons. Nets of SV2 labelled endings were coincident with NFH labelled nets particularly at the mid‐atrial region and at the superior and inferior cavo‐atrial junctions (Figure 3). Fine SV2 labelled fibres appeared to extend from end‐net formations and most of these SV2 labelled fibres did not co‐label for NFH. With manipulation of the fine focus, it was discernible that SV2 labelled fibres were closer to the endocardial surface than neurofilament labelled fibres. Across all atrial whole mounts, no flower‐spray endings were observed. Also, no obvious motor varicosities were observed. In the teased lumbrical preparations, differential labelling of axons was also present with SV2 clearly labelling the vesicles of terminal boutons whilst NFH labelled axonal projections leading to end plates (Figure 3d,e).

### 3.3 Synaptophysin labelling

**Figure 4:**
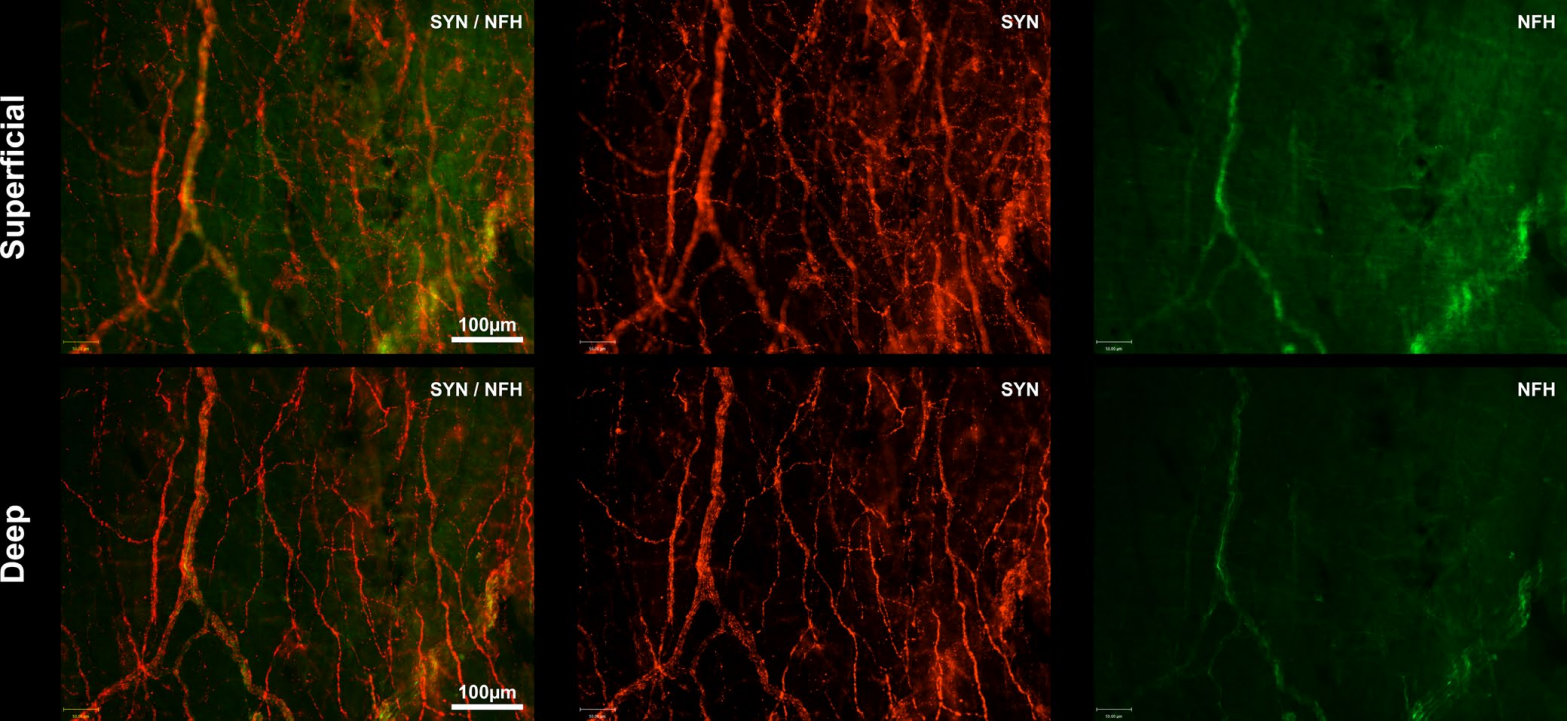
NFH and SYN expression in the subendocardial neural plexus. Shown are superficial and deep views of the endocardium which were imaged at the superior cavo‐atrial junction. SYN is shown in red and NFH in green. An end‐net fibre network (SYN) is evident. SYN was expressed both in small superficial and large deep fibres whilst NFH was confined to large axon bundles in the deeper layers of the endocardium. NFH, neurofilament heavy chain; SYN, synaptophysin

**Figure 5:**
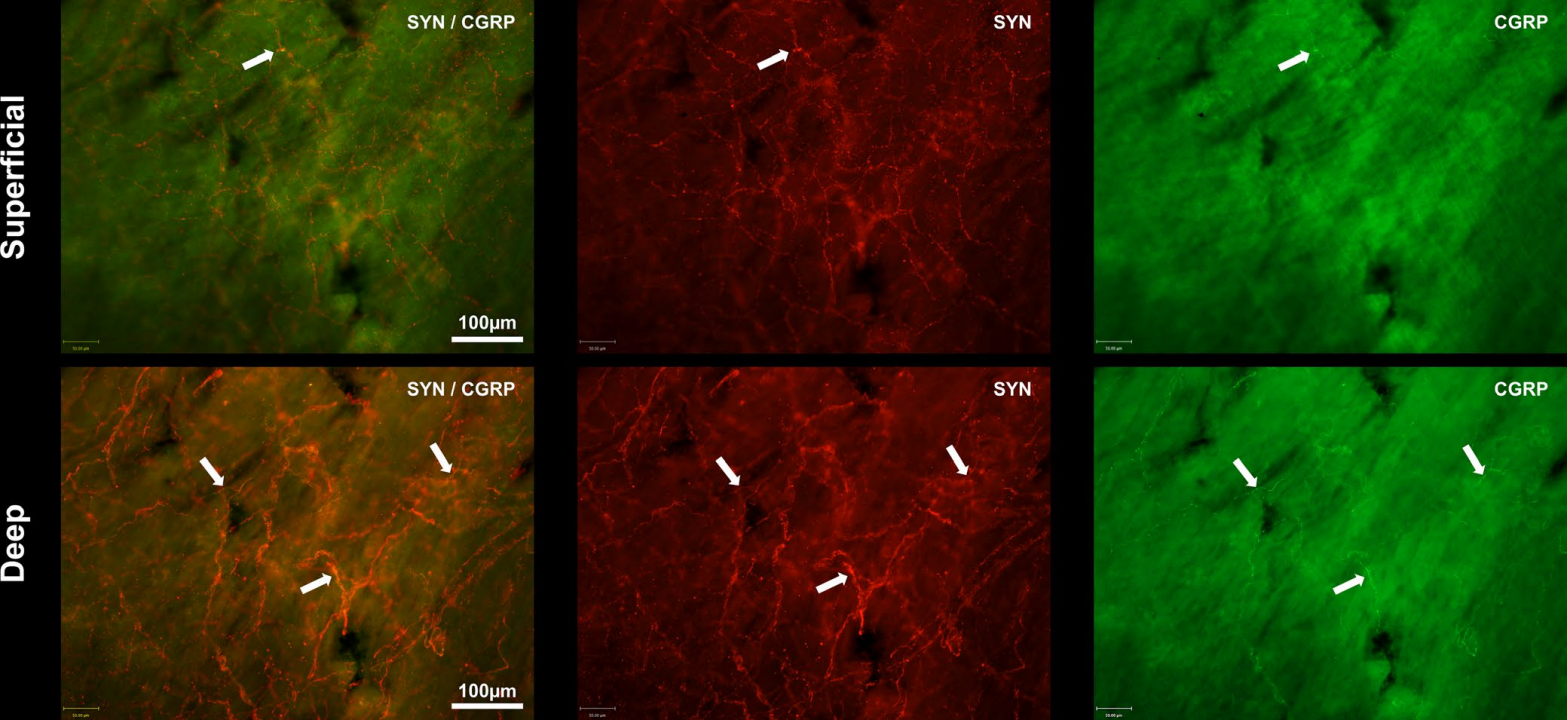
CGRP and SYN expression in the subendocardial neural plexus. Shown are superficial and deep views of the endocardium which were imaged at the superior cavo‐atrial junction. SYN is shown in red and CGRP in green. A SYN‐labelled end‐net is present within superficial and deep layers of the endocardium. CGRP was much less prevalent in the superficial than deep layer and was observed within isolated single fibres or in SYN‐labelled axon bundles. White arrows indicate axons labelled with CGRP. CGRP, calcitonin gene‐related peptide; SYN, synaptophysin

**Figure 6:**
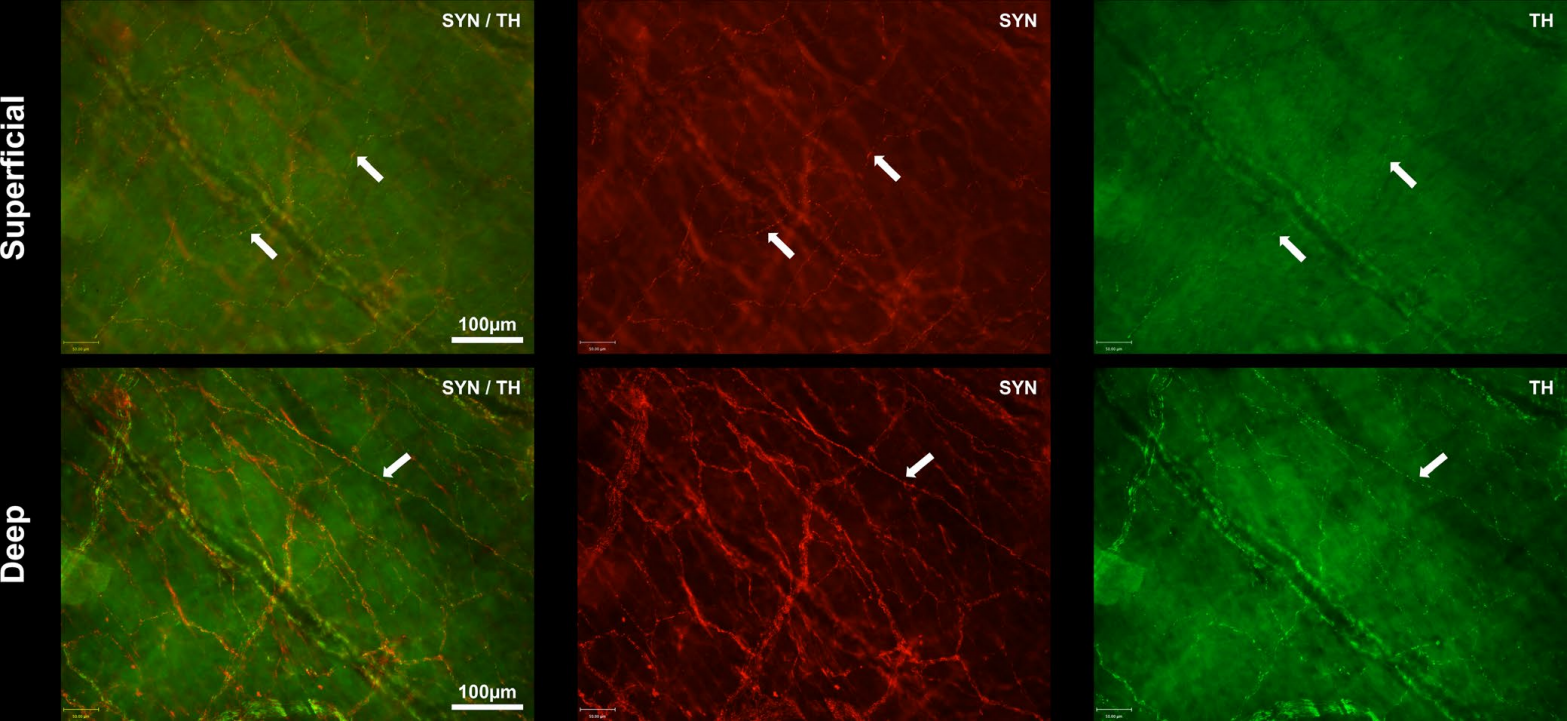
TH and SYN expression in the subendocardial neural plexus. Shown is a superficial and deep view for TH/SYN. SYN is shown in red and TH in green. Both SYN and TH labelled an end‐mesh network visible in both superficial and deep layers. In the superficial layer, the white arrows indicate the location of a fine open end‐net formation. TH co‐localised and compartmentalised with the SYN‐labelled fibres (see white arrow in the deep layer). Overall, TH labelling was less abundant than SYN. SYN, synaptophysin; TH, tyrosine hydroxylase

**Figure 7:**
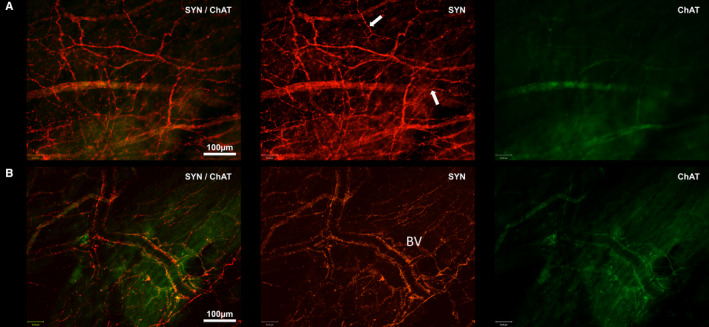
ChAT and SYN expression in the subendocardial neural plexus. Shown are two deep views of ChAT/SYN labelling at the endocardium of the superior cavo‐atrial junction. Two deep views are presented as no ChAT labelling was observed in the superficial endocardial layer. SYN is shown in red and ChAT in green. (a) SYN‐labelled end‐net fibre network (white arrows). (b) SYN‐labelled blood vessel. ChAT labelling is sparse and courses within nerve bundles or along blood vessel walls. BV, blood vessel; ChAT, choline acetyltransferase; SYN, synaptophysin

**Figure 8:**
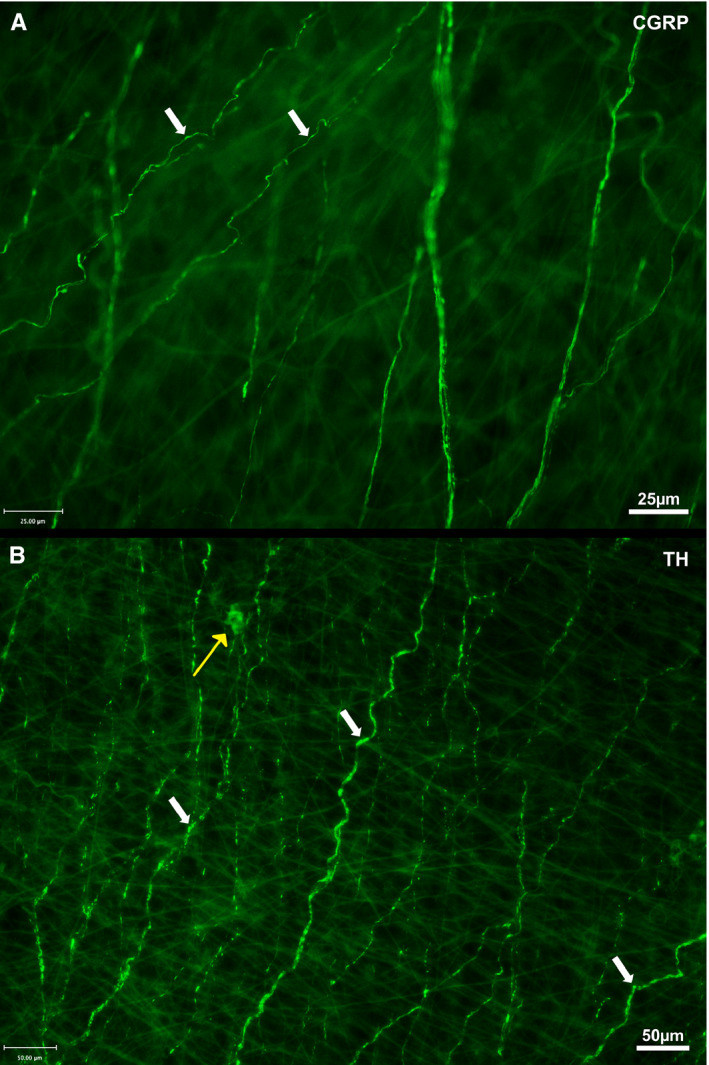
Distinctive “wavy” single fibres at the inferior cavo‐atrial junction. Shown are endocardial views of tissues from different animals. Immunohistochemical labelling revealed distinctive “wavy” single fibres running parallel to each other and perpendicular to the direction of blood flow. These fibres (white arrows) were observed for CGRP (a), TH (b), SV2 and SYN (not shown). A cell soma is present (yellow arrow) and could be that of a SIF cell. CGRP, calcitonin gene‐related peptide. SIF, small intensely fluorescent; SV2, synaptic vesicle protein 2; SYN, synaptophysin; TH, tyrosine hydroxylase

Synaptophysin labelling was widespread and abundant (Figures 4, 5, 6, 7, 8). The walls of blood vessels in the endocardium were densely innervated with SYN‐positive endings (Figures 5, 6, 7). On the adluminal surface (superficial), there was a distinctive loose network of single SYN‐labelled fibres, most apparent at the caval entrances to the atrium. Synaptophysin immunoreactivity was also evident on distinctive, “wavy” single fibres running parallel to each other and perpendicular to the direction of blood flow. Beneath the adluminal network, fibres were in bundles of different sizes as well as occurring singly (Figure 6).

### 3.4 Calcitonin gene‐related peptide and neurofilament labelling

There was less abundant labelling throughout the entire wholemount with either CGRP or NFH antibodies. Neurofilament heavy polypeptide immunoreactivity was confined to large axon bundles in the endocardium (Figure 4). Calcitonin gene‐related immunoreactivity (IR) was present on single fibres which occurred alone or in amongst larger SYN‐positive axon bundles (Figure 5). The larger axon bundles were not present in the superficial, adluminal layer. In thin walls of the IVC, CGRP‐IR often coincided with SYN labelling on the “wavy” fibres running around the circumference of the vessel. Many of the larger bundles containing CGRP labelled fibres appeared to be either innervating or running alongside blood vessels, whereas NFH labelled fibres were not present on blood vessels.

### 3.5 Tyrosine hydroxylase labelling

Tyrosine hydroxylase co‐labelled SYN reactive axon bundles and single fibres in all locations, including those on blood vessels (Figure 6). The single fibres were fine and varicose, and this was especially apparent both within the IVC, where they ran in parallel lines as described above for the SYN reactive fibres; and in the loose, open network evident within the superficial adluminal endocardial layer. However, the TH and SYN labelling did not precisely co‐localise. Tyrosine hydroxylase positive structures were observed in amongst SYN‐positive structures within axon bundles, usually being fewer in number (Figure 6). Even on single fibres, as in the loose superficial endocardial network, the labelling appeared compartmentalised (Figure 6).

### 3.6 Choline acetyltransferase labelling

Choline acetyltransferase labelling was far less prevalent than SYN/TH labelling. It was not present in the superficial endocardial layer but was present on large bundles of axons below the level of the endocardium (Figure 7a). Positive ChAT labelling was also observed on blood vessel walls and in surrounding nerve bundles (Figure 7b) below the level of the adluminal endocardium.

### 3.7 Innervation of the inferior cavo‐atrial junction

Over the inferior IVC there were distinctive, “wavy”, single fibres running in parallel with one another. These fibres coursed around the circumference of the vessel, that is perpendicular to the direction of blood flow. Antibodies directed against CGRP, TH (Figure 8) and SYN/SV2 (not shown) labelled these fibres clearly.

### 3.8 Methylene blue labelling

**Figure 9:**
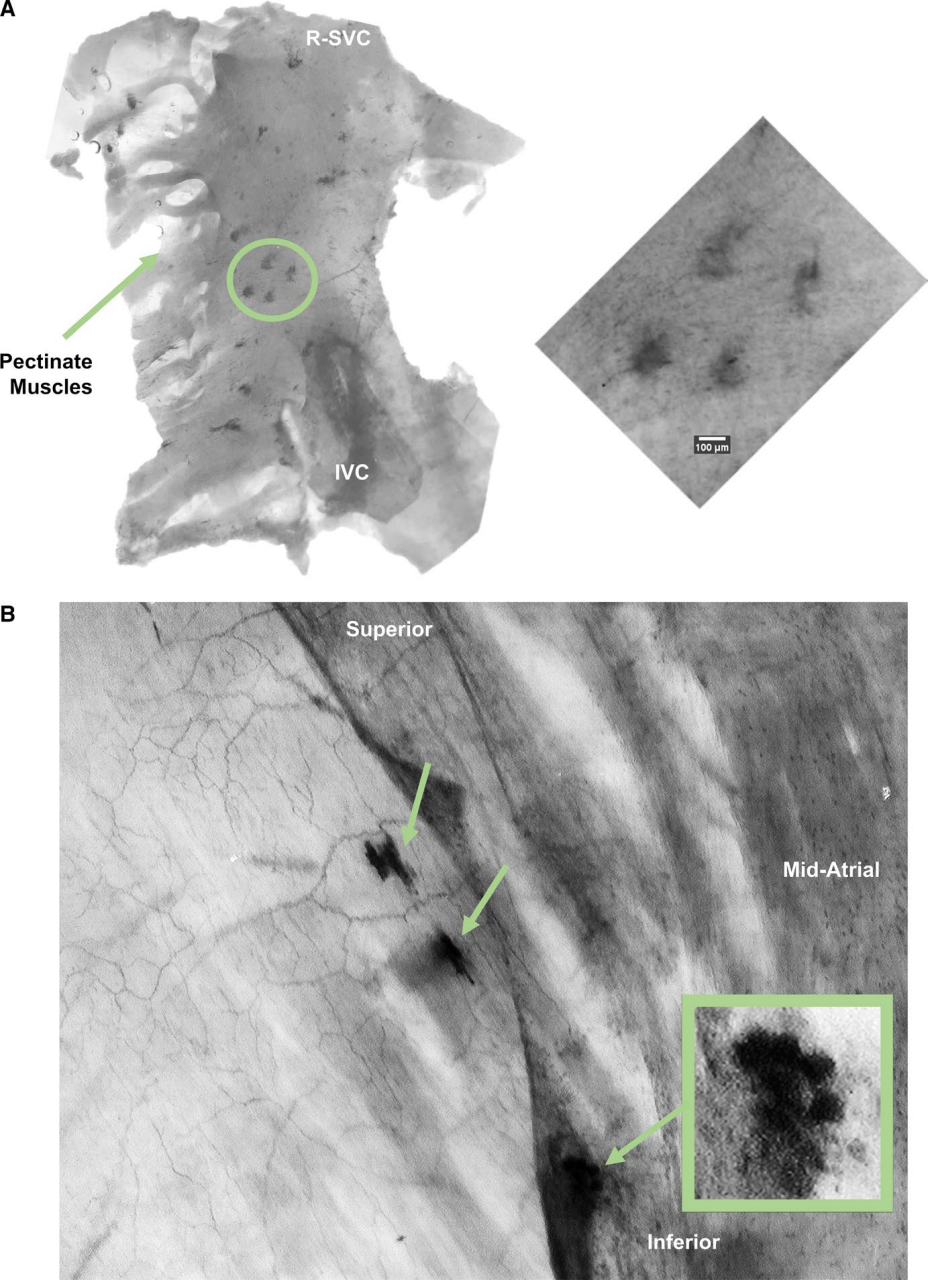
Methylene blue labelled structures in the right atrium of the rat. (a) View of the endocardial surface of the mid‐atrial region. In each whole mount approximately 20–30 discrete areas of intense methylene blue labelling were observed. Inset is a high‐power view of four such structures which have a diameter of approximately 100 μm consistent with flower‐spray endings. (b) Endocardial surface of the right atrial whole mount. An end‐net is visible on the left which straddles two flower‐spray endings. Inset is a contrast‐enhanced view of a flower‐spray ending the morphology of which is consistent with terminals labelled with anterograde tracing techniques. Flower‐spray endings were difficult to image but were observed most clearly visualised with transillumination. As (b) was imaged using non‐calibrated imaging apparatus, scale bar is not included. IVC, inferior vena cava; R‐SVC, right superior vena cava

Twenty to thirty discrete areas of intense methylene blue uptake (consistent with flower‐spray endings) were observed in each atrial whole mount. These were primarily located between the hourglass and the pectinate muscles of the auricle and were approximately 100 μm in diameter (Figure 9a). End‐net formations were observed across the entire whole mount and appeared most dense at the superior cavo‐atrial junction. Flower‐spray endings were difficult to image clearly which could reflect that they are deeper than the end‐net. One flower‐spray ending was suspended in a leaflet of the Eustachian valve and thus was more easily imaged than other endings. A contrast‐enhanced view of this ending revealed that it had a multilobulated structure (Figure 9b).

## 4 DISCUSSION

This study has examined the architecture of the cardiac mechanoreceptors present in endocardium of the right atria of the rat. All the labelling techniques adopted consistently revealed end‐net structures. The structures labelled by SV2 antibodies were consistent with those elicited by SYN. These structures were found within the subendocardium around the entrances of the great veins to the atria. We propose that some end‐nets are sensory fibres and argue that because deformation of the atrial wall during atrial filling and contraction occurs three dimensionally, these structures can accurately reflect this to the brain.

### 4.1 Complex unencapsulated nerve endings/flower‐sprays

The flower‐spray atrial mechanoreceptor type was only revealed following methylene blue staining. Transillumination was adopted to obtain clear images of these endings. The protocol consistently labelled 20–30 small structures primarily located between the hourglass and pectinate muscles. The structures identified by methylene blue uptake are putatively identified as flower‐sprays because they are irregularly shaped and approximately 100 μm in diameter. This size is consistent with the flower‐spray endings reported in rodent anterograde tracing experiments (Cheng *et al.*, 1997) and with those described (50–350 μm) using methylene blue labelling in other species such as the dog, cat, monkey, lamb, rabbit and guinea pig (Coleridge *et al.*, 1957; Miller and Kasahara, 1964; Linden and Kappagoda, 1982). It was difficult to image an afferent axon emerging from these endings; however, it is reasonable to assume that our imaging may be unable to capture these as they are reportedly 4–6 μm in diameter (Linden and Kappagoda, 1982).

The combined number of flower‐spray endings found in both the left and right atria has been studied in dogs, cats, monkeys and lambs and varies from 150 to 300 (Holmes, 1957a; Miller and Kasahara, 1964) with approximately one third being present in the right atrium (Linden and Kappagoda, 1982). Our observations pertaining to the number of flower‐spray endings in the right atrium of the rat are in keeping with these estimates given its smaller size. It is possible that the number we report is an underestimation related to the methodological approach adopted.

Nearly all descriptions since Berkley (1895) and Smirnow (1895) using heavy metal silver and methylene blue staining report extensive complex unencapsulated terminals and end‐net formations in the atrial endocardium. Methylene blue staining and DiI appear to consistently localise complex end‐organs and end‐net formations (Linden and Kappagoda, 1982; Cheng *et al.*, 1997). Whilst the antibody markers SYN, CGRP, SV2 and NFH failed to localise flower‐spray endings, they consistently revealed the end‐net formations. Therefore, the ability to detect these structures may depend on staining/methodological regime employed. However, manipulation of antibody concentration/incubation duration, alteration of permeabilisation agent conditions and adjusting tissue clearance techniques did not label flower‐sprays. It is possible that flower‐spray endings are located deeper than the end‐net and if this is the case then perhaps despite the adjustments the antibodies did not penetrate the tissue sufficiently. Irrespective of whether flower‐spray endings are deeper than the end‐net, it should also be considered that flower‐spray endings may not express NFH, SYN, CGRP or SV2 and thus were not labelled with the utilised immunohistochemical techniques.

### 4.2 End‐net structure

Synaptophysin CGRP, SV2 and NFH, whether as single or in combination consistently revealed a similar innervation pattern of the end‐net structure within the endocardium. Synaptophysin is a protein found in synaptic vesicles and as such has been frequently used to identify synapses. However, synaptic‐like vesicles have been commonly noted in mechanosensitive nerve terminals for many decades (Katz, 1966) and these vesicles also express synaptophysin (de Camilli *et al.*, 1988). Sensory fibres have been suggested to be negative for SYN, a marker of presynaptic vesicles unless they are varicose. The SYN and CGRP labelling we report did have varicose terminals suggestive of sensory fibres in the endocardium, although CGRP labelling was infrequent compared to SYN. Neurofilament is utilised mainly for identifying myelinated endings (Lawson and Waddell, 1991; Molliver *et al.*, 1995; Henry *et al.*, 2012). Neurofilament labelling revealed a rich neural plexus just below the level of the adluminal endocardium, a pattern consistent with those previously observed with anterograde labelling techniques (Cheng *et al.*, 1997). Interestingly, varicosities were not observed in these deeper structures, which suggest that the observed neurofilament labelling may be sensory in nature.

The end‐net mesh was most dense at the superior and inferior cavo‐atrial regions. Stereological quantification further confirmed and qualified this by suggesting the subendocardial neural plexus innervation density is greater for the superior cavo‐atrial junction compared to the mid‐atrial region. This corresponds with the early descriptions of atrial receptors elicited from functional data arising from the cat and dog and other species including humans (Johnston, 1968) indicating a rich distribution around the vein‐atrial junctions (Coleridge *et al.*, 1973; Linden and Kappagoda, 1982).

With both SYN and SV2, over some areas of the inner endocardial surface, the net consisted of single fibres. Small beaded SYN/SV2 labelled fibres and coarser NFH labelled fibres consistently demonstrated the end‐net to repeatedly divide and coalesce, forming bundles of axons which presumably project to the vagal nerve and towards the brain. Also, with NFH the consistent hourglass arrangement of axons in the posterior atrial wall was revealed. The inter‐caval region of the rabbit right atrium has been studied with cholinesterase/silver staining and similarly an hourglass arrangement of axons has been described (Roberts, 1991). In the rat right atrium, we have observed that an upper hourglass limb (anatomical left) consistently tracks towards the root of the cardiac vagal branch and as such we hypothesise that this hourglass structure could be vagal in origin. Whether the end‐net is sensory in nature has not been confirmed. However, one study (Coleridge *et al.*, 1957) identified the location of receptors following physiological stimulation and recording of subsequent action potentials. The location of the receptors was confirmed by methylene blue staining and revealed end‐nets as well as complex unencapsulated endings. This suggest the potential for the end‐net being a sensory structure.

### 4.3 Synaptophysin & calcitonin gene‐related peptide

Calcitonin gene‐related peptide, commonly used as a biomarker for chemosensitive afferents and peptidergic pain‐sensing C‐fibres (Kopp *et al.*, 2001; Russell *et al.*, 2014), was far less prevalent. We found CGRP expressed in fine varicose endings which appeared singly or within larger bundles of SYN‐labelled fibres. It was also most frequently in the endocardial layer nearest the myocardium rather than the adluminal surface, and this is in agreement with our previous findings (Shenton and Pyner, 2014). Although CGRP is often considered a marker of unmyelinated nerves (Kakudo *et al.*, 1988; Alvarez *et al.*, 1991; Bickel *et al.*, 1999; Ishikawa *et al.*, 2005), the distinction is not always categoric. For example, whilst investigating the rat vagal nerves, Kakudo *et al.* (1988) found CGRP immunoreactivity in 5% of myelinated axons, whereas 50% of unmyelinated axons expressed CGRP. Although fewer in number, some of the CGRP positive myelinated fibres found in the vagus may have a role in stretch sensitivity and control of ingestive behaviour of the oesophagus (Andrew, 1956; Rodrigo *et al.*, 1985).

### 4.4 Neurofilament & synaptic vesicle protein 2

Synaptic vesicle protein 2 was used to try and identify vesicle‐rich mechanotransductive regions within the right atrium. The fourth lumbrical muscle served as a positive control and no annulospiral endings were labelled. However, axons in motor end plates were differentially labelled with NFH whilst SV2 labelled discrete vesicles of the terminal bouton. We attribute the lack of annulospiral labelling to inadequate disruption and permeabilisation of the spindle capsule during the tissue preparation process. In the atrial whole mounts, extensive co‐localisation of NFH and SV2 was noted in larger fibres however fine fibres which extended from end‐net formations labelled for SV2 almost exclusively. Synaptic vesicle protein 2 labelled fibres were observed across the entirety of whole mount samples but were noted to be most dense at the cavo‐atrial junctions. No flower‐spray endings were observed. The SV2 labelling approach may also have labelled post‐ganglionic motor fibres. It should be noted that SV2 labelled fibres were not observed to possess varicosities and were located closer to the endocardial surface than NFH labelled end‐nets which is at odds with the supposition that these fibres are motor in nature. To better elucidate areas of vesicle recycling and identify mechanically sensitive atrial receptors, future studies could use vesicular glutamate transporter (vGluT) family which has been implicated in mechanotransduction. Merkel cells have been shown to express vGluT1, vGluT2 and vGluT3 (Nunzi, Pisarek, and Mugnaini, 2004), and recently, vGluT1 has been used to label proprioceptive sensory terminals in the muscle spindle and Golgi tendon organ of the mouse (de Nooij *et al.*, 2015). It has also been described that incubation with glutamate increases discharge rate from the muscle spindle (Banks *et al.*, 2002). With this evidence it is reasonable to investigate the role of the vGluT family in mechanically sensitive atrial receptors.

### 4.5 Distinguishing between sensory and motor fibres

Whilst SYN, CGRP, NFH and SV2 have labelled fibres, it cannot be stated with certainty that the observed neuronal structures are truly sensory even if they resemble the end‐net as described in the literature. The presence of motor varicosities (or lack thereof) is also an inadequate means of differentiation. Therefore, the additional targets of ChAT and TH in combination with SYN were examined. Choline acetyltransferase is expressed in acetylcholine‐releasing motor neurons (Kou *et al.*, 1995) and anti‐ChAT antibodies would label parasympathetic efferent neurons present in the atria. Tyrosine hydroxylase is expressed in sympathetic motor fibres (Cheah and Geffen, 1973) and anti‐TH antibodies would label catecholamine‐releasing sympathetic motor fibres present in the atria.

### 4.6 Synaptophysin & tyrosine hydroxylase

Within the fine, superficial end‐nets labelled with SYN and TH, it is probable that the same fibres were labelled and that the internal concentration of SYN and TH is variable and compartmentalised. In large bundles of fibres, TH and SYN also appeared compartmentalised and may have been on separate fibres however higher magnification confocal imaging would be required to precisely discriminate between fibres. Electron microscopy from mini‐pigs indicates efferent sympathetic nerve fibres course with the afferent fibre to end near the receptor ending (Tranum‐Jensen, 1975, 1979). In zebra fish larvae, TH fibres described as efferents have been shown to play a role in modulating sensory nerve endings (Haehnel‐Taguchi *et al.*, 2018). The exclusive TH‐positive fibre terminals we describe projecting in the endocardium may be sympathetic efferents and may release monoamines locally for yet unknown purposes. Tyrosine hydroxylase is a marker of catecholaminergic neurones, where the neurotransmitter is adrenaline, noradrenaline or dopamine and is commonly used to identify sympathetic efferents (Foss *et al.*, 2015) although there is evidence for TH expression on mechanosensitive afferents (Vyas *et al.*, 2017; Wu *et al.*, 2018).

The finding that the majority of SYN‐labelled endings also expressed TH was a surprise since TH is usually considered as a marker of sympathetic efferents. Tyrosine hydroxylase/SYN‐labelled endings have also been reported in human right atrial material (Marron *et al.*, 1995; Bohlender *et al.*, 2018). With SYN or TH as co‐markers, Bohlender described abundant angiotensin fibre innervation of human right atrium. The TH and SYN fibres were varicose with a comparable anatomical distribution. The TH fibres were mostly SYN‐positive. Bohlender assumed these fibres to be efferent, but as alluded to above this may not be the case. In the right cavo‐atrial junction we have shown that the SYN‐labelled terminals in the endocardium (Shenton and Pyner, 2014) have a morphology characteristic of sensory endings (Drummond *et al.*. 1998; Maeda *et al.*, 1999). Tyrosine hydroxylase has been described in sensory afferents, both chemosensory (Katz *et al.*, 1987; Czyzyk‐Krzeska *et al.*, 1991; Finley *et al.*, 1992) and, more recently, mechanosensory (Vyas *et al.*, 2017; Wu *et al.*, 2018). Tyrosine hydroxylase fibres could be dopaminergic and dopaminergic mechanosensory endings have been reported in *C. elegans* (Han *et al.*, 2017) and these endings also expressed the mechanosensory TRP‐4 channel. Furthermore, the nematode TRP‐4 channel is very similar to mammalian transient receptor potential vanilloid 4 (TRPV4), which we have found previously in cavo‐atrial endocardium of the rat heart coinciding with SYN labelling (Shenton and Pyner, 2014).

### 4.7 Synaptophysin & choline acetyltransferase

A minority of SYN‐labelled nerves were co‐labelled with ChAT (a marker of parasympathetic efferents) and these were large bundles of axons below the adluminal level of the endocardium, suggesting the preponderance of SYN immunoreactivity was indeed on afferents.

### 4.8 Intrinsic innervation of the atria

Our investigations in whole mounts have focussed on the subendocardial nerve plexus, whereas previous wholemount studies, in both rats (Richardson *et al.*, 2003) and mice (Rysevaite *et al.*, 2011; Li *et al.*, 2014) have examined the epicardial surface. Intracardiac ganglia (ICG) are found within the epicardial tissue and in rats these form a ring‐like plexus around the entry of the pulmonary veins (Richardson *et al.*, 2003). We imaged the endocardial aspect of the right atrium and hence the ICG were not readily visible through the relatively thick myocardium. Nevertheless, there remains a possibility that we have imaged some components of the intrinsic innervation of the atria, especially with regard to the ChAT labelling. Richardson *et al.* (2003) found all intracardiac neurons contained immunoreactivity to ChAT and neuropeptide Y (NPY). By contrast, whilst nerve fibres around the ganglia occasionally expressed TH, the neuronal somata themselves were never positive for this marker; although it did strongly label small intensely fluorescent cells (Huber, 2006) associated with the ganglia. Around the ganglia TH labelled nerve fibres did not express SYN, whereas broadly in agreement with our own observations, TH‐positive fibres present in the myocardium and blood vessel walls always co‐expressed SYN. This could be suggestive of a distinct functional role for these dual labelled endings. Similarly, none of these three studies (Richardson *et al.*, 2003; Rysevaite *et al.*, 2011; Li *et al.*, 2014) found CGRP in the cell bodies of the ICG.

### 4.9 Functional considerations

The exact nature of the stimulus which activates cardiac mechanoreceptors remains unknown. It is likely to be a combination of chemical and mechanical stimuli. In rodent muscle spindles stretch initiates the opening of stretch sensitive channels and the sensitivity of the mechanosensitive endings is modified by glutamate released from the endings themselves (Bewick et al., 2005; Bewick and Banks, 2015). Other mechanoreceptors are likely to share similar features, although the detail may vary. Whilst this may describe the transduction of a stimulus, the precise nature of these stimuli remains elusive. However, three‐dimensional deformation during atrial filling and contraction changes the curvature and thus tension of the wall and may offer a mechanism to initiate the transduction process. Our study revealed a dense network of putative sensory fibres arranged in an end‐net mesh in the region where the caval veins enter the atria. This region undergoes large changes in dimensions and tension and this end‐net mesh may be the mechanism by which these dynamic variables are communicated to the brain.

The presence of SYN/CGRP/TH axons at the adluminal side of the endothelium may indicate a chemosensing role for these fibres. It stands to reason that because of their close proximity to the blood‐filled atrial lumen, they could be receptive to gases and molecules that diffuse into the paracellular space. Some authors report most atrial afferent fibres to be chemosensitive (Waldmann *et al.*, 2006). Fine endocardial terminals protruding between endothelial cells into the atrial lumen, which are likely to be chemosensitive, have been reported in human cadaver endothelium (Bohlender *et al.*, 2018). Furthermore, these were either angiotensin II/SYN or TH/SYN positive.

## 5 SUMMARY

The subendocardial nerve nets were found to be denser at the superior cavo‐atrial junction than the mid‐atrial region. The rat heart has a bicuspid Eustachian valve which is richly invested with nerve fibres. Adluminal networks had the finest diameters and stained positively for synaptic vesicles (SV2 and SYN), CGRP and TH. These plexuses may represent sympathetic post‐ganglionic fibres and/or sensory afferents. The latter are candidate substrates for volume receptors which are depolarised by myocyte contraction in atrial systole or excited by stretch during atrial filling. Deeper nerve fibres appeared coarser and may be cholinergic (positive staining for ChAT). Flower‐spray endings were never observed using immunohistochemistry but were delineated clearly with the intravital stain methylene blue. This suggests that either flower‐spray endings do not express NFH, SYN, SV2, TH, CGRP or ChAT or that antibody access to these endings was restricted. Thus, future directions need to address the anatomic relationship of the nerve fibres to the cardiac muscle with the discharge characteristics of atrial receptors reflecting differing receptor type.

## CONFLICT OF INTEREST

None declared.

## AUTHOR CONTRIBUTIONS

The authors take responsibility for the integrity of the data and accuracy. All authors contributed to conceptualisation and experimental design, data analysis, manuscript drafting and approval of the submitted manuscript. SYN, NFH, TH, ChAT and CGRP labelling, imaging and analysis was performed by Dr. Fiona Shenton and Dr. Susan Pyner. NFH, SV2 and methylene blue imaging and analysis was performed by Dr. Thomas Campbell and Prof. James Jones.

## Supporting information

Appendix S1

## Data Availability

The data that support the findings of this study are available from the corresponding author upon reasonable request.
