# Supplementary material for: Distribution and morphology of sensory and autonomic fibres in the subendocardial plexus of the rat heart
Source: J Anat. 2020 Aug 11;238(1):36–52. doi: 10.1111/joa.13284 (PMC7754995; doi:10.1111/joa.13284)
Supplement: Supplementary file 1 — Appendix S1 [file JOA-238-36-s001.docx]

**Appendix S1**

**Calculation of length area density (L_A_) per unit area and coefficient of error**

As per Mouton (2011), the formula for length area density (L_A_) per unit area was defined as:

L_A_ = (π/2) × [(Total Number of Intersections) / (Total Probe Length)]

The coefficient of error was defined as the standard error of the mean of repeated estimates divided by the mean (source: MBF Bioscience webpage (<http://www.stereology.info/coefficient-of-error/> , access date 13/01/2020).

**Tissue Preparation & Immunohistochemical Protocols Flowchart**

**Method One**(University College Dublin)

**Method Two**

(Durham University)


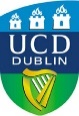

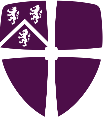


Tissue Blocked for 30 minutes at 4°C.
[Blocking solution constituents: 1% (w/v) BSA, 5% (v/v) NGS, 1 x PBS]

**Abbreviations:** BSA, Bovine Serum Albumin. CGRP, Calcitonin Gene-Related Peptide. ChAT, Choline Acetyltransferase. DPX, Distyrene Plasticizer Xylene. NDS, Normal Donkey Serum. NFH, Neurofilament Heavy Chain. NGS, Normal Goat Serum. OCT, Optimal Cutting Temperature compound. PB, Phosphate Buffer. PBS, Phosphate Buffered Saline. SYN, Synaptophysin. SV2, Synaptic Vesicle Protein 2. TH, Tyrosine Hydroxylase.

Tissue fixed & permeabilised in precooled
(-20°C) methanol for 30 minutes

Tissue fixed 4% (w/v) paraformaldehyde overnight at 4°C

Tissue rinsed with 0.1M PB

Tissue incubated with primary antibodies in 0.1M PB for 48 hours. [of anti-SYN and either anti-CGRP, anti-NFH, anti-ChAT or anti-TH]. See table 1 for dilutions.

Prior to imaging, tissue triple rinsed in 1 x PBS. Each rinse lasted 10 minutes and was performed in light protected humidity chamber at 4°C.

Tissue incubated in primary antibody diluent (1:500 chicken anti-NFH / 1:25 mouse anti-SV2 in 1% BSA, 5% NGS, 1 x PBS)

Tissue rinsed in 1 x PBS

Tissue triple rinsed with 1% (w/v) BSA in 1 x PBS. Each rinse 10 min duration.

Tissue incubated with secondary antibodies diluent in light protected humidity chamber at 4°C overnight. (1:250 goat anti-chicken Alexa Fluor ® 568 / 1:100 goat anti-mouse Alexa Fluor ® 488)

Prior to imaging, tissue triple rinsed in 1 x PBS (each rinse 10 mins), mounted in OCT on a glass slide and cover slipped.

Tissue blocked with 10% (v/v) NDS in 0.1M PB (30 mins)

Tissue permeabilised in Triton-X-100 for 45 mins [0.1% (v/v) in 0.1M PB]

Tissue was triple rinsed in 0.1M PB

Tissue incubated with secondary antibodies in 0.1M PB (2 hours at 20°C, see table 1 for dilutions)

Tissue triple rinsed in 0.1M PB, spread onto charged slides and dried overnight

Prior to imaging, tissue was dehydrated in series of alcohols (2 mins in 50%, 70%, 95%, 70%, 50%), mounted in DPX and cover slipped.
